# Heterogeneous information network based clustering for precision traditional Chinese medicine

**DOI:** 10.1186/s12911-019-0963-0

**Published:** 2019-12-19

**Authors:** Xintian Chen, Chunyang Ruan, Yanchun Zhang, Huijuan Chen

**Affiliations:** 1 School of Computer Science, Fudan University, Shanghai, China; 2 College of Engineering and Science, Victoria University, Melbourne, Australia; 3 Cyberspace Institute of Advanced Technology, Guangzhou University, Guangzhou, China; 4 School of Basic Medical Science, Shanghai University of Traditional Chinese Medicine, Shanghai, China

**Keywords:** TCM, Formula, Heterogeneous Information network, Clustering, Ranking

## Abstract

**Background:**

Traditional Chinese medicine (TCM) is a highly important complement to modern medicine and is widely practiced in China and in many other countries. The work of Chinese medicine is subject to the two factors of the inheritance and development of clinical experience of famous Chinese medicine practitioners and the difficulty in improving the service capacity of basic Chinese medicine practitioners. Heterogeneous information networks (HINs) are a kind of graphical model for integrating and modeling real-world information. Through HINs, we can integrate and model the large-scale heterogeneous TCM data into structured graph data and use this as a basis for analysis.

**Methods:**

Mining categorizations from TCM data is an important task for precision medicine. In this paper, we propose a novel structured learning model to solve the problem of formula regularity, a pivotal task in prescription optimization. We integrate clustering with ranking in a heterogeneous information network.

**Results:**

The results from experiments on the Pharmacopoeia of the People’s Republic of China (ChP) demonstrate the effectiveness and accuracy of the proposed model for discovering useful categorizations of formulas.

**Conclusions:**

We use heterogeneous information networks to model TCM data and propose a TCM-HIN. Combining the heterogeneous graph with the probability graph, we proposed the TCM-Clus algorithm, which combines clustering with ranking and classifies traditional Chinese medicine prescriptions. The results of the categorizations can help Chinese medicine practitioners to make clinical decision.

## Background

Traditional Chinese medicine(TCM) has a long history and is one of the oldest forms of medicine. The fact that traditional Chinese medicine can exist for thousands of years is a proof that TCM has the value of its medical form. Traditional Chinese medicine is being accepted by the public. More and more researchers are working on Chinese medicine, and more Chinese medicines are used in different countries.[1].

The same disease may have different symptoms in different patients. Due to the differences between patients, accurate diagnosis and treatment are even more important[2]. In the field of Western medicine, doctors explore the cause of the disease and focus on treating specific parts of the body. However, Chinese medicine works differently. Traditional Chinese medicine and Western medicine have fundamental differences in diagnosis and treatment. The Chinese medicine practitioner explores the internal and external causes of the patient and combines them accordingly.

**Fig. 1:**
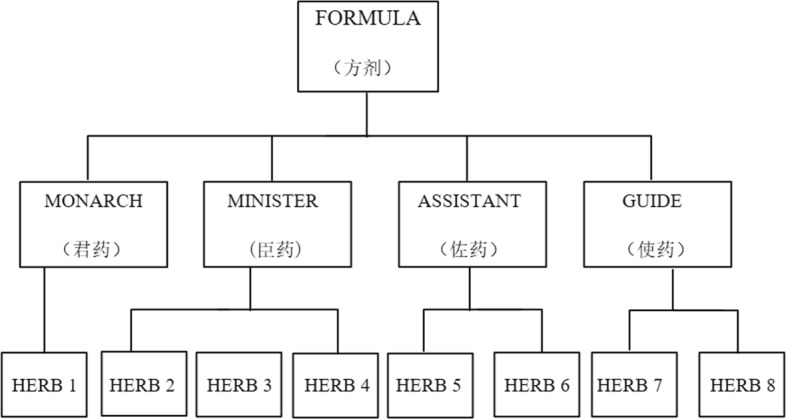
The composition of a formula

In TCM, herbal remedies are usually based on traditional Chinese medicine formula, and the use of only one type of herbal medicine rarely occurs. Each herb has its advantages and disadvantages, and they are formulated in a reasonable proportion. Figure 1 is a schematic diagram of the composition of a formula [20]. Formula is the foundation of traditional Chinese medicine and the core of TCM research. Traditional Chinese medicine data such as ancient Chinese literature and clinical prescriptions contain prescription data. How to study prescription data through scientific and technical analysis has become the main topic of TCM informatization. Traditional Chinese medicine data generally appears in the form of texts with strong natural language, usually characterized by unstructured, massive and heterogeneous. These characteristics have become a huge challenge in the process of informationization of Chinese medicine. In order to meet the challenge, how to integrate the data of heterogeneous Chinese medicine and model representation, using the data processing method to analyze Chinese medicine data has become an important work of TCM informationization.[3].

The traditional model can no longer meet the inheritance and development of TCM knowledge. The main bottleneck is the organization form of the knowledge and the limitation of human resources. Simply, TCM knowledge mainly exists in the forms of prescriptions, medical records, etc. Unlike ordinary texts, TCM texts have irregular natural language, which creates great difficulties for digitization. At the same time, because the degree of informationization of TCM is not high, the inheritance model of TCM is generally an apprenticeship mode in which an old practitioner cultivates apprentices. As a result, some knowledge cannot be passed down in time. How to pass on the vast knowledge of TCM in an efficient way has become a hot topic in the field of TCM research.

The development of machine learning and artificial intelligence is conducive to drive the inheritance of traditional Chinese medicine. Experience can be regarded as a kind of knowledge in artificial intelligence, which can be used as input data for machine learning. Secondly, "dialectical treatment" is the basic principle of TCM diagnosis of diseases. This is in line with the basic principles of machine learning: the model is trained based on the training set, and the model gives the target value based on the input values. Xu et al. used data mining methods to explore drug combinations for nonalcoholic fatty liver disease[4]. Chen et al. used a three-part map to explore the symptom-disease pattern in the case[5]. Liu et al. used CRF to learn the characteristic patterns in TCM cases to identify symptoms and cases[6]. Wang et al. proposed a probabilistic model for the analysis of symptoms, diseases, and drug relationships in TCM cases[7]. Although some of these tasks can also learn the low-dimensional representation of nodes, they focused on data with rich semantics. The majority of TCM medical data, particularly formula-based prescriptions, are lack of good semantic information.

In this paper, we propose a clustering algorithm based on probability model to solve the clustering problem of heterogeneous information network of traditional Chinese medicine. For a given target type, we aim to generate the clustering of the target object and the ranking information of the objects in the cluster. We propose a heterogeneous information network of traditional Chinese medicine, which is a star network schema. The algorithm can obtain stable clustering results after many iterations. Our main contributions are as follows:

We propose a clustering algorithm based on probability model, which integrates clustering with ranking information for Chinese medicine formula categorization and discover potential knowledge. The algorithm can help doctors optimize diagnosis and prescription. According to the ranking information of each object in the cluster, doctors can easily assess its importance.

We conducted experiments on real data sets of traditional Chinese medicine. The experimental results show that the algorithm is effective and accurate. The algorithm can provide reasonable clustering results for optimizing prescriptions and is confirmed by Chinese medicine experts.

Social networks, the Internet, medical information networks and many other networks in real world contain a large number of interconnected nodes. These networks are called information networks [8]. The ubiquitous information network is an important part of modern information infrastructure. The nodes in the information network are connected by an intricate network structure, which contains rich information. At present, information network analysis is not only widely concerned by researchers in various fields, but also a hot topic in the field of data mining and information retrieval. However, most information network related research has a basic assumption: the types of nodes and the types of links in the network are unique. That is to say, the researcher does not distinguish the types of nodes and regards them as homogeneous information networks, for example, the author collaboration network. In fact, these networks are full of different kinds of nodes, and it is more reasonable to think of them as heterogeneous information networks (HINs) with different types of nodes and links. Heterogeneous information networks contain richer semantic information in nodes and links. For example, in a bibliographic information network, papers are connected to each other by different types of nodes, such as authors, conferences, and topics. If a paper is connected to two authors at the same time, the two authors have a cohesive relationship with this paper[9].

Ranking is an important task on the heterogeneous information network, and it faces some challenges. First, there are different types of objects and relationships in HIN. Second, different types of objects and relationships have different semantic information. In addition, the ranking information of different objects will affect each other.Taking the bibliographic heterogeneous network as an example, ranking on authors may have different results under different meta paths [10] since these meta paths will construct different link structures among authors. Moreover, the rankings of different-typed objects have mutual effects. For example, reputable authors generally publish papers in top journals[11].

Clustering is a process of classifying similar objects. The objects in the same cluster are similar, and the objects between different clusters are dissimilar. Traditional clustering is generally based on object-based features, such as the K-means algorithm. At present, network-based clustering (community discovery) and other issues are receiving widespread attention. The correlation model usually treats it as a homogeneous information network and divides the network into a series of subgraphs in a given way (e.g., normalized cuts and modularity). Many algorithms have been proposed to solve this NP-hard problem, such as the spectral method [12], greedy method and sampling technique [13]. Some studies consider both the link information and attribute information of the object to improve clustering accuracy [14]. Further, clustering on heterogeneous information networks has received attention.

Unlike homogeneous networks, different types of objects on heterogeneous information networks present a huge challenge to the task.

On the one hand, different types of objects in the network bring new forms of clustering. For example, a cluster may contain different types of objects with the same topic. A cluster of database domains contains authors, conferences, and papers in this field. In this case, clustering on heterogeneous information networks has richer semantics, but it also faces more challenges. On the other hand, the rich information contained in the network helps to improve the accuracy of the task. Li et al. put forward the SCHAN algorithm to solve the clustering problem in Attributed HIN[15]. Zhou et al. designed a dynamic learning algorithm SI-Cluster for social influence based graph clustering[16]. Luo et al. introduced the concept of relation-path to measure the similarity between two objects and propose a framework for semi-supervised learning in HINs[17]. Undoubtedly, these approaches improved the clustering performance, but they were confined to entities with rich attributes or labeled data.

## Methods

### Problem formulation

In this section, we introduce several important concepts and define the problem of clustering in the TCM HIN.

#### Definition 1 (Heterogeneous Information Network).

An information network is defined as an undirected graph *G*=<*V*,*E*> with an object type mapping function *τ*:*V*→*T* and link type mapping function *ψ*:*E*→*R*, where $T=\{T_{k}\}_{k=1}^{|T|}$ is a set of object types and $R=\{R_{k}\}_{k=1}^{|R|}$ is a set of link types on T. Specifically, we call such an information network a HIN when |*T*|≥2 and a homogeneous information network when |*T*|=1.

#### Definition 2.

(Network schema) Given a HIN *G*=<*V*,*E*>, a network schema is defined as an undirected graph *S*_*G*_=<*T*,*R*>, where $T=\{T_{k}\}_{k=1}^{|T|}$ is a set of object types and $R=\{R_{k}\}_{k=1}^{|R|}$ is a set of link types on T.

#### Definition 3 (Star Network).

**Fig. 2:**
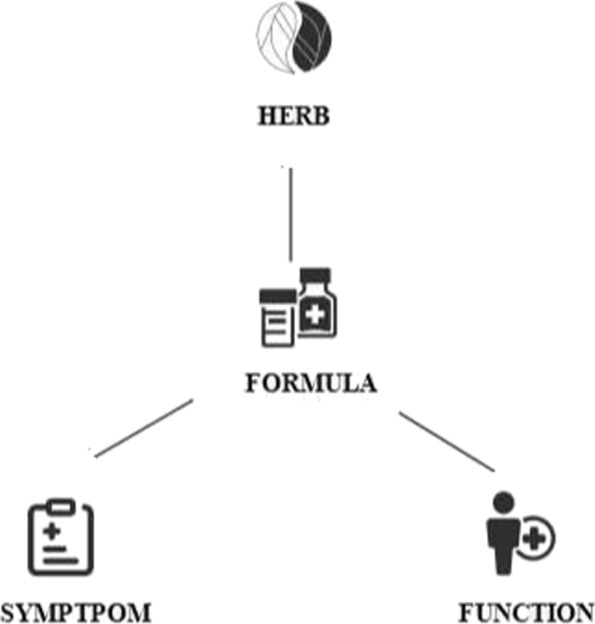
Schema for TCM-HIN

A HIN *G*=<*V*,*E*> on |*T*| types of entities $T=\{T_{k}\}_{k=0}^{K}(K\ge 2)$ is with a star network schema if, ∀*e*=<*t*_*i*_,*t*_*j*_>∈*E*,*t*_*i*_∈*T*_0_∧*t*_*j*_∈*T*_*k*_(*k*≠0), or vise versa. G is then called a star network. *T*_0_ is called the target type, and *T*_*k*_(*k*≠0) are called attribute types[11].The schema for TCM-HIN is shown in Fig. 2.

In this paper, we use *T* to represent the set of types of TCM entities. We have *T*={*F*_*m*_,*F*_*c*_,*H*,*S*}, where *F*_*m*_,*F*_*s*_,*H*, and *S* denote the entity types “formula”, “function”, “herb”, and “symptom”, respectively. For convenience, we use *F*_*m*_ to denote both the set of objects belonging to the “formula” type and the type name. Other types are similar to *F*_*m*_. We use *R*={*F*_*m*_*F*_*c*_,*F*_*m*_*H*,*F*_*m*_*S*} to represent the set of types of TCM relations on T, where *F*_*m*_*F*_*c*_,*F*_*m*_*H*, and *F*_*m*_*S* denote the relation types “formula-function”, “formula-herb”, and “formula-symptom”, respectively.

#### Definition 4 (TCM-HIN).

**Fig. 3:**
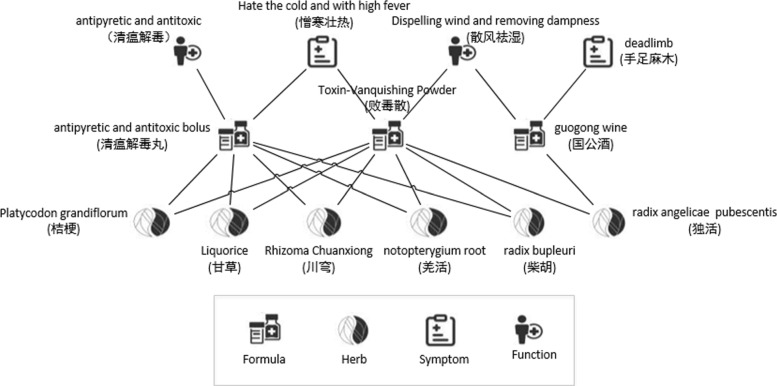
An example of TCM-HIN

TCM-HIN is a HIN *G*=<*V*,*E*> with star network schema *S*_*G*_=(*T*,*R*), where *T*={*F*_*m*_,*F*_*c*_,*H*,*S*} and *R*={*F*_*m*_*F*_*c*_,*F*_*m*_*H*,*F*_*m*_*S*}[18]. An example of TCM-HIN is shown in Fig. 3.

Based on these definitions, we can formulate our key problem as follows: given a TCM-HIN *G*=<*V*,*E*>, the target type *T*_0_, and a specified cluster number *K*, we aim to generate *K* clusters {*C*_*K*_} for target objects from target type on *G*, as well as the within-cluster ranking information for all the objects based on these clusters in the network.

We propose a ranking-based clustering algorithm for mining formula categorization. In this section, we first introduce the overall clustering framework. Then, we explain four important parts of the algorithm in detail.

### Framework of algorithm

To integrate ranking with clustering in a HIN, a model is required to flexibly support these two tasks. Therefore, we propose a probabilistic generative model to estimate the probability of target and attribute objects in the network. We can use the rankings of objects to infer the probability of objects and clustering information. The major difficulty in clustering in a HIN is the definition and calculation of pairwise similarity between objects. We map each target object into a low-dimensional space defined by the current clustering result to avoid defining and calculating similarity between each pair of objects.

TCM clustering is mainly composed of the following five steps:

∙ Step 0: Randomly initialize partitions of target objects and induce initial clusters from the original network according to these partitions, i.e., $\{C_{k}^{0}\}_{k=1}^{K}$. Decompose the star schema network into three bipartite networks, where *V*={*F*_*m*_,*S*},{*F*_*m*_,*H*}, and {*F*_*m*_,*S*}, respectively.

∙ Step 1: For each bipartite network, build a ranking-based probabilistic generative model for target type and attribute type, i.e., $\{P(x|C_{k}^{t})\}_{k=1}^{K}$.

∙ Step 2: For each bipartite network, estimate the posterior probabilities to each cluster for each target object, i.e., $\{P(C_{k}^{t}|x)\}_{k=1}^{K}$.

∙ Step 3: Calculate the distance from each target object to each cluster center based on the posterior probabilities and then assign each target object to the nearest cluster.

∙ Step 4: Repeat Steps 1, 2 and 3 until the cluster does not change significantly or the iteration number is larger than a predefined number.

**Fig. 4:**
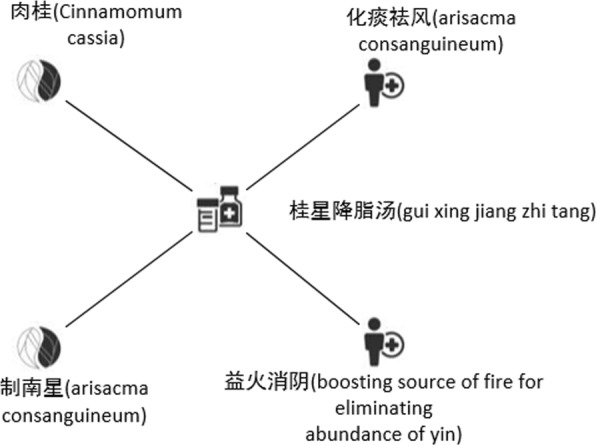
Schema for TCM-HIN


The core framework of TCM-Clus is shown in Algorithm 1. In TCM-HIN, a formula may connect to more than one herb, function, and symptom. For example, in Fig. 4, a formula called  contains two herbs called (Cinnamomum cassia) and (arisacma consanguineum) and has two functions called (dispelling pathogenic wind and eliminating phlegm) and (boosting source of fire for eliminating abundance of yin). However, it does not mean that (Cinnamomum cassia) has both functions. Therefore, we should decompose the TCM-HIN into several bipartite networks as above, instead of simply making estimation in original TCM-HIN[11]. In this paper, we decompose the TCM-HIN into three bipartite networks $\phantom {\dot {i}\!}(G_{S},G_{H},G_{F_{c}})$, which are induced graphs of the original graph G. Because the ranking function and posterior probability estimation for each bipartite network are similar, we only present the explanation for the bipartite network *G*_*S*_=<*V*,*E*>, where *V*={*F*_*m*_,*S*} and *G*_*S*_⊆*G*.

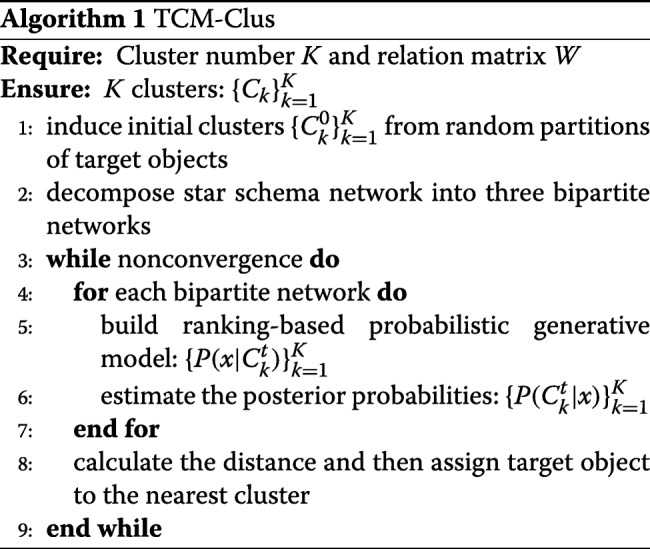


### Ranking function

In information network analysis, the two most important ranking algorithms are PageRank [19] and HITS [20], both of which are successfully applied to Internet searches. PageRank is a link analysis algorithm that assigns a numerical weight to each object of the information network, with the purpose of “measuring” its relative importance within the object set. Conversely, HITS ranks objects based on two scores: authority and hub. Authority estimates the value of the content of the object, whereas hub measures the value of its links to other objects. Both PageRank and HITS evaluate the static quality of objects in the information network, which is similar to the intrinsic meaning of our ranking methods. However, both PageRank and HITS are designed on a network of webpages, which is a directed homogeneous network, and the weight of the edge is binary.

#### Definition 5 (Ranking Distribution and Ranking Function).

A ranking distribution *P*(**T**) on a type of object T is a discrete probability distribution, which satisfies *P*(*T*=*t*)≥0(∀*t*∈*T*) and $\sum _{t \in T}P(T=t)=1$. A function *f*:*G*→*P*(**T**) defined on an information network *G* is called a ranking function on type *T* if given an information network *G*, it can output a ranking distribution *P*(**T**) on *T*.

Ranking is beneficial for people to grasp the importance of objects in a collection. For example, PageRank and authority of HITS represent the static importance of webpages, while the rank of a document to a given query in text retrieval reflects the relevance of the document to that query.

We use W to represent the adjacency matrix, which we call the relation matrix, between the target type and the attribute type. We can define the matrix as

$$W_{F_{m}S}(i,j)=p_{ij} $$

where *i* and *j* are two objects from type *F*_*m*_ and type *S* and *p*_*ij*_ is the frequency of *i* that links to *j*.

We have two simple empirical rules:

∙ Rule 1: Highly ranked formulas can cure highly ranked symptoms.

∙ Rule 2: One highly ranked symptom can enhance the rank of another symptom if they are cured by the same formula.

According to Rule 1, we generate the ranks of types *F*_*m*_ and *S* as follows:

1 $$P(f_{mi}|F_{m},G)=\sum_{j=1}^{|S|}W_{F_{m}S}(f_{mi},j)P(s_{j}|S,G)  $$

2 $$P(s_{j}|S,G)=\sum_{i=1}^{|F_{m}|}W_{SF_{m}}(s_{j},i)P(f_{mi}|F_{m},G)  $$

where *G* is a network, *f*_*mi*_ is an object from type *F*_*M*_, and *s*_*j*_ is an object from type *S*. Notice that the normalization will not change the ranking position of an object, but it provides a relative importance score to each object. After normalization, we have

3 $$ P(\mathbf{F}_{\mathbf{m}}|F_{m},G)=\frac{W_{F_{m}S}P(\mathbf{S}|S,G)}{\| W_{F_{m}S}P(\mathbf{S}|S,G)\|}   $$

4 $$ P(\mathbf{S}|S,G)=\frac{W_{SF_{m}}P(\mathbf{F}_{\mathbf{m}}|F_{m},G)}{\| W_{SF_{m}}P(\mathbf{F}_{\mathbf{m}}|F_{m},G)\|}   $$

We can prove that *P*(**F**_**m**_|*F*_*m*_,*G*) is the eigenvector of $W_{F_{m}S}W_{SF_{m}}$ and *P*(**S**|*S*,*G*) is the eigenvector of $W_{SF_{m}}W_{F_{m}S}$.

#### *Proof*

Combining (3) and (4), we can obtain

$$\begin{aligned} P(\mathbf{F}_{\mathbf{m}}|F_{m},G) &=\frac{W_{F_{m}S}P(\mathbf{S}|S,G)}{\| W_{F_{m}S}P(\mathbf{S}|S,G)\|}\\ &=\frac{W_{F_{m}S} \frac{W_{SF_{m}}P(\mathbf{F}_{\mathbf{m}}|F_{m},G)}{\| W_{SF_{m}}P(\mathbf{F}_{\mathbf{m}}|F_{m},G)\|}} {\| W_{F_{m}S}\frac{W_{SF_{m}}P(\mathbf{F}_{\mathbf{m}}|F_{m},G)}{\| W_{SF_{m}}P(\mathbf{F}_{\mathbf{m}}|F_{m},G)\|}\|}\\ &=\frac{W_{F_{m}S}W_{SF_{m}}P(\mathbf{F}_{\mathbf{m}}|F_{m},G)}{\| W_{F_{m}S}W_{SF_{m}}P(\mathbf{F}_{\mathbf{m}}|F_{m},G)\|} \end{aligned} $$

Thus, *P*(**F**_**m**_|*F*_*m*_,*G*) is the eigenvector of $W_{F_{m}S}W_{SF_{m}}$. Similarly, *P*(**S**|*S*,*G*) is the eigenvector of $W_{SF_{m}}W_{F_{m}S}$. We can use the power method to calculate the primary eigenvector. □

When considering Rule 2, we can revise the equation as

5 $$ \begin{aligned} P(s_{j}|S,G)&=\alpha\sum_{i=1}^{|F_{m}|}W_{SF_{m}}(s_{j},i)P(f_{mi}|F_{m},G)\\ &+(1-\alpha)\sum_{i=1}^{|S|}W_{SS}(j,i)P(s_{j}|S,G) \end{aligned}  $$

where $\phantom {\dot {i}\!}W_{SS} = W_{SF_{m}}W_{F_{m}S}$ and parameter *α*∈[0,1] determines the weight of “symptom-formula” and “symptom-symptom”. Similarly, we can prove that *P*(**S**|*S*,*G*) should be the primary eigenvector of $\alpha W_{SF_{m}}W_{F_{m}S}+(1-\alpha)W_{SS}$, and *P*(**F**_**m**_|*F*_*m*_,*G*) should be the primary eigenvector of $\alpha W_{F_{m}S}(I-(1-\alpha)W_{SS})^{-1}W_{SF_{m}}$.

In fact, if we consider the problem from the perspective of the meta path, these two rules reflect the meta path based relationship between objects. Rule 1 corresponds to meta path *S*−*F*_*m*_, while Rule 2 corresponds to meta path *S*−*F*_*m*_−*S*.

### Ranking-based probabilistic generative model

We assume that the probabilities that objects from different types will be visited in the given network are independent of each other. The probability of visiting an object in *G* can be decomposed into two parts:

$$p(x|G)=p(T_{x}|G)\times p(x|T_{x},G) $$

where the first part *p*(*T*_*x*_|*G*) is the general probability that the type of *x* will be visited in the network *G* and the second part *p*(*x*|*T*_*x*_,*G*) is the probability that an object *x* will be visited among all the objects from type *T*_*x*_ in the network *G*. Here, we consider the ranking distribution as the probability of objects to be visited within their own type in a given information network G. We will show that the value of *p*(*T*_*x*_|*G*) is not important and can be set to 1 later. In a subnetwork *G*_*k*_=*G*(*C*_*k*_), we can calculate the probability of visiting an object:

$$p(x|G_{k})=p(T_{x}|G_{k})\times p(x|T_{x},G_{k}) $$

However, we will encounter problems if we use the above equation directly. In a given cluster, a target object may link to objects whose ranking is zero in that cluster. In addition, a target object may not belong to the current cluster. If we simply assign the probability of visiting the target object as zero in that cluster, then we will lose some important information. To solve this problem, we can use smoothing, which is a well-known technique in information retrieval to cope with the zero probability problem for missing terms in a document [21]. We add the global ranking to smooth the conditional ranking before calculating the visibility for the target object:

6 $$ p(x|T_{x},G_{k})=(1-\lambda)p(x|T_{x},G_{k}) + \lambda p(x|T_{x},G)  $$

where the smoothing parameter *λ* denotes the portion of global ranking.

To evaluate the model, we make another independence assumption that the probabilities that objects from the same types will be visited are also independent of each other:

7 $$ p(x_{i},x_{j}|T_{x},G)=p(x_{i}|T_{x},G)\times p(x_{j}|T_{x},G)  $$

where *x*_*i*_,*x*_*j*_∈*T*_*x*_.

### Posterior probability estimation using EM algorithm

To determine which cluster target objects belong to, we estimate the posterior probability for each target object. For convenience, we use *X* and *Y* to represent types *F*_*m*_ and *S*, where |*X*|=*m* and |*Y*|=*n*.

Given a clustering on the input network G, we can calculate the posterior probability for each target object using the Bayesian rule:

$$p(G_{k}|x_{i})\propto p(x_{i}|G_{k})\times p(k) $$,

where *p*(*x*_*i*_|*G*_*k*_) is the probability that target object *x*_*i*_ will be visited in cluster *k* and *p*(*k*) denotes the relative size of cluster *k*. From this formula, we can see that type probability *p*(*T*|*G*) is just a constant for calculating the posterior probabilities for target objects and can be neglected.

Let *Θ* be the parameter matrix, which is an *m*×*K* matrix: *Θ*_*m*×*k*_={*P*(*G*_*k*_|*x*_*i*_)}(*i*=1,2,⋯,*m*;*k*=1,2,⋯,*K*). To obtain the best *Θ* that maximizes the likelihood to generate the whole bipartite network, we have the following likelihood function:

$$L(\Theta|W_{XY})=P(W_{XY}|\Theta)= \prod_{i=1}^{m}\prod_{j=1}^{n}P(x_{i},y_{j}|\Theta)^{W_{XY}(i,j)}$$,

where *P*(*x*_*i*_,*y*_*j*_|*Θ*) is the probability of generating link <*x*_*i*_,*y*_*j*_> given the current parameter. Because it is difficult to maximize *L* directly, we apply the EM algorithm to solve the problem. In the E-Step, we introduce hidden variable *z*∈{1,2,⋯,*K*} to represent the cluster label that a link <*x*,*y*> is from. The complete log likelihood can be written as

$$\begin{aligned} \log L &=\log \prod_{i=1}^{m}\prod_{j=1}^{n}P(x_{i},y_{j},z|\Theta)^{W_{XY}(i,j)} \\ &=\log \prod_{i=1}^{m}\prod_{j=1}^{n}[p(x_{i},y_{j}|z,\Theta)p(z|\Theta)]^{W_{XY}(i,j)} \\ &=\sum_{i=1}^{m} \sum_{j=1}^{n} W_{XY}(i,j) \log (p(x_{i},y_{j}|z)p(z|\Theta)) \end{aligned} $$

Initially, we can set the parameters in *Θ*^(0)^ as even values. The expectation of the log likelihood under the current distribution of *Z* is

8 $$ \begin{aligned} &Q(\Theta,\Theta^{(t)})\\ &=\sum_{k=1}^{K}\sum_{i=1}^{m}\sum_{j=1}^{n}[W_{XY}(i,j)\\ &\quad\times \log(P(x_{i},y_{j}|z=k)P(z=k|\Theta^{(t)}))P(z=k|x_{i},y_{j},\Theta^{(t)})]\\ &=\sum_{i=1}^{m}\sum_{k=1}^{K}\sum_{j=1}^{n}[W_{XY}(i,j) \log(P(z=k|\Theta^{(t)}))P(z=k|x_{i},y_{j},\Theta^{(t)})]\\ &\quad+\sum_{k=1}^{K}\sum_{i=1}^{m}\sum_{j=1}^{n}[W_{XY}(i,j)\log(P(x_{i},y_{j}|z=k))P(z=k|x_{i},y_{j},\Theta^{(t)})] \end{aligned}  $$

where *Θ*^(*t*)^ is the parameter matrix after *t* iterations.

We can use the Bayesian rule to calculate conditional distribution *P*(*z*=*k*|*x*_*i*_,*y*_*j*_,*Θ*^(*t*)^) as follows:

9 $$ {}P(z=k|x_{i},y_{j},\Theta^{(t)})\propto p^{(t)}(x_{i}|k)p^{(t)}(y_{j}|k)p^{(t)}(z=k)   $$

In the M-Step, to obtain *P*^(*t*+1)^(*z*=*k*) that maximizes *Q*(*Θ*,*Θ*^(*t*)^), we introduce the Lagrange multiplier *λ*. For each *P*(*z*=*k*), where *k*=1,2,⋯,*K*, we have

$${}\begin{aligned} &\frac{\partial}{\partial P(z=k)}[Q(\Theta,\Theta^{(t)})+\lambda(\sum_{k=1}^{K}P(z=k)-1))]=0\\ \Rightarrow&\sum_{i=1}^{m}\sum_{j=1}^{n}W_{XY}(i,j)\frac{1}{P(z=k)}P(z=k|x_{i},y_{j},\Theta^{(t)})+\lambda=0 \end{aligned} $$

Now, integrating with (9), we can obtain the new estimation for *P*(*z*=*k*):

10 $$ {}p^{(t+1)}(z=k)=\frac{\sum_{i=1}^{m} \sum_{j=1}^{n} W_{XY}(i,j)P\left(z=k|x_{i},y_{j},\Theta^{(t)}\right)}{\sum_{i=1}^{m} \sum_{j=1}^{n} W_{XY}(i,j)}  $$

Finally, each parameter in *Θ* is calculated as

11 $$ P(G_{k}|x_{i})=P(z=k|x_{i})=\frac{P(x_{i}|G_{k})P(z=k)}{\sum_{l=1}^{K}P(x_{i}|G_{l})P(z=l)}  $$

### Cluster assignment

After we obtain the estimations for each target object in each bipartite network, we can represent the target object as a 3*K*-dimensional vector

12 $$\begin{array}{*{20}l} {}\vec{s}_{X_{i}}&=(p_{S}(G_{1}|x_{i}),\ldots,p_{S}(G_{K}|x_{i}),\ldots,\\ &\qquad p_{F_{c}}(G_{K}|x_{i}),\ldots,p_{H}(G_{K}|x_{i}))\end{array} $$

The centers for each cluster can thus be calculated accordingly, which is the arithmetic mean of $\vec {s}_{X_{i}}$ for all *x*_*i*_ in each cluster:

13 $$\vec{s}_{C_{k}}=\frac{\sum_{x\in C_{k}}\vec{s}(x)}{|C_{k}|} $$

where *x*_*i*_ is an object from type *F*_*m*_ and |*X*_*k*_| is the size of the cluster k.

The distance between an object and cluster is defined by 1 minus cosine similarity:

14 $$ D(x,C_{k})=1-\frac{\sum_{l=1}^{K}\vec{s}_{x}(l)\vec{s}_{C_{k}}(l)}{\sqrt{\sum_{l=1}^{K}(\vec{s}_{x}(l))^{2}}\sqrt{\sum_{l=1}^{K}(\vec{s}_{C_{k}}(l))^{2}}}  $$

Then, we can assign each object to the cluster with the smallest distance.

### User-guided clustering

User guidance is critical for clustering objects in the network[22]. Using different types of link information in a network, different reasonable clustering results can be generated. We take user guidance in the form of object seeds for some clusters as the prior knowledge for the clustering result *Θ* by modeling the prior as a Dirichlet distribution rather than treating them as hard labeled ones. For each target object *x*_*i*_, its clustering probability vector *P*(**G**|*x*_*i*_) is a multinomial distribution, which is generated from some Dirichlet distribution. If *x*_*i*_ is labeled as a seed in cluster *k*^∗^,*P*(**G**|*x*_*i*_) is then modeled as being sampled from a Dirichlet distribution with parameter vector $\phantom {\dot {i}\!}\lambda _{d}\mathbf {e}_{k^{*}}+1$, where $\phantom {\dot {i}\!}\mathbf {e}_{k^{*}}$ is a K-dimensional basis vector, with the *k*^∗^th element as 1 and 0 elsewhere. If *x*_*i*_ is not a seed, *x*_*i*_ is then assumed as being sampled from a uniform distribution, which can also be viewed as a Dirichlet distribution with a parameter vector of **1**.The density of *P*(**G**|*x*_*i*_) given such priors is

$${}P(\mathbf{G}|x_{i},\lambda_{d})=\left\{ \begin{aligned} &\prod_{k}P(G_{k}|x_{i})^{\mathbf{1}\{ x_{i} \in G_{k} \}\lambda_{d}}&,x_{i} is \,labeled\, as\, k^{*}\\ &1&,x_{i}\, is\, not \,labeled. \end{aligned} \right. $$

where **1**{*x*_*i*_∈*G*_*k*_} is an indicator function, which is 1 if *x*_*i*_∈*G*_*k*_ holds and 0 otherwise. The hyperparameter *λ*_*d*_ is a nonnegative value and controls the strength of users’ confidence over the object seeds in each cluster.

### Time complexity analysis

The time complexity of TCM-Clus is composed of the following parts. First, the time complexity for ranking is *O*(*t*_1_|*E*|), where *t*_1_ is the iteration number and |*E*| is the number of links. Notice that |*E*|≪|*V*|^2^ in a sparse network, where |*V*| is the total number of objects in the network. Second, for the posterior probability estimation, we need to calculate *O*(*K*|*E*|+*K*+*mK*) parameters at each iteration, where the time complexity for (9) is *O*|*K*|*E*||, the time complexity for (10) is *O*(*K*), and the time complexity for (11) is *O*(*mK*). Third, the cluster adjustment for each object has complexity *O*(*mK*^2^). Since we need to compute the distance between each object and each cluster, the dimension of an object is *K*. In total, the time complexity for TCM-Clus is *O*(*t*_1_|*E*|+*t*_2_(*K*|*E*|+*K*+*mK*)+*mK*^2^), where *t*_2_ is the iteration number of the estimation. If the network if sparse, which is typical in most applications, the time complexity is almost linear to the number of objects in the network.

## Results

In this section, we conduct several experiments to show the effectiveness of TCM-Clus. We discuss the evaluation of TCM-Clus. First, we introduce the datasets used in this paper. Then, we discuss the evaluation of TCM-Clus.

### Datasets

In this paper, we use the real datasets ChP, The Pharmacopoeia of the People’s Republic of China 2015 Edition (http://wp.chp.org.cn/en/index.html), and 3K+ TCM clinical cases mainly in the stomach. We use herb information in Volume I, which contains 2598 types of medicinal materials without classifications, to set up our experiments. ChP is a unstructured corpus and contains various information. We only extract formula, function, herb, and symptom to build TCM-HIN.

### Quantitative evaluation

We use FVIC (fraction of vertices identified correctly) to evaluate the clustering accuracy of the clustering results. It has been used in many research projects and is defined as follows:

15 $$ \begin{aligned} &olSet(c,c*)=\{v|v\in c\wedge v\in c^{*}\}\\ &maxolSet(c,C_{K})=max_{c\in C_{K}}\{|olSet(c,c^{*})|\}\\ &FVIC=\sum_{c\in C_{F}}\frac{maxolSet(c,C_{K})}{N} \end{aligned}  $$

where *C*_*F*_ and *C*_*K*_ represent the found clusters and known clusters, respectively. *c* and *c*^∗^ are clusters in *C*_*F*_ and *C*_*K*_, respectively. N is the number of objects in the network. FVIC evaluates the average matching degree by comparing each predicted cluster with the most matching real cluster. A higher score indicates a better clustering with respect to the ground truth.

We compare TCM-Clus with spectral clustering, which is the k-way Ncut algorithm and has been used to cluster Western medical records[23]; PaReCat, which has been used to cluster Chinese medical records for the task of patient record categorization[24]; and K-Means, a common clustering technique. In this experiment, we fix the smoothing parameter *λ* as 0.2 and weight parameter *α* as 0.8. The accuracy results are shown in Table 1.

**Table 1:** Clustering Accuracy for Two Datasets

	K-Means	PaReCat	spectral	TCM-Clus
Chp	0.473	0.748	0.741	0.825
Clinical cases	0.589	0.835	0.787	0.875

We can observe that TCM-Clus achieves the best clustering accuracy on the two datasets. K-Means shows poor performance because our medical data lack semantic information. Spectral has a good result. However, due to omitting the structure of the graph, it has worse performance compared to TCM-Clus. The performance of PaReCat is closest to our algorithm, but it is more suitable for patient record categorization with disease, symptom and herb. We have shown that TCM-Clus can indeed improve clustering accuracy by integrating ranking with clustering.

### Parameter study

**Fig. 5:**
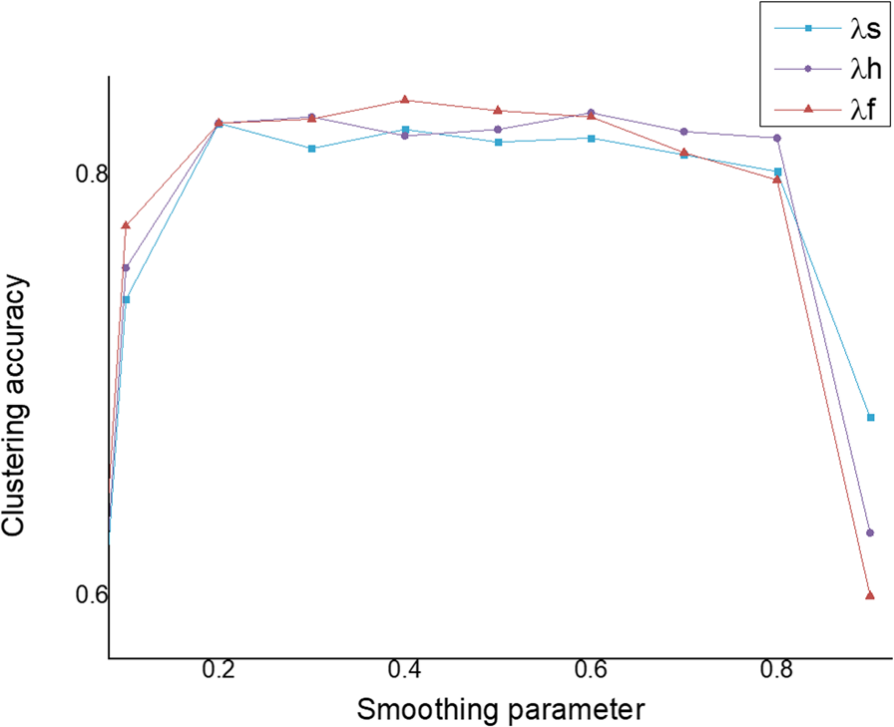
Clustering accuracy with different smoothing parameters

We use clustering accuracy to analyze the effect of different smoothing parameters *λ* on Chp dataset. We represent three different *λ*s for symptom, herb, function as *λ*_*s*_,*λ*_*h*_ and *λ*_*f*_, respectively. We change one type of *λ* and fix the other two to 0.2. We run TCM-Clus on ChP datasets, and the results are shown in Fig. 5. The results are based on ten different initial partitions. We can observe that TCM-Clus achieves better accuracy when *λ* is from 0.1 to 0.8. If the smoothing parameter *λ* is too small or too large, it means that we only consider conditional ranking or global ranking. Too small (*λ*→0) or too large (*λ*→1) will decrease the performance of TCM-Clus.

**Fig. 6:**
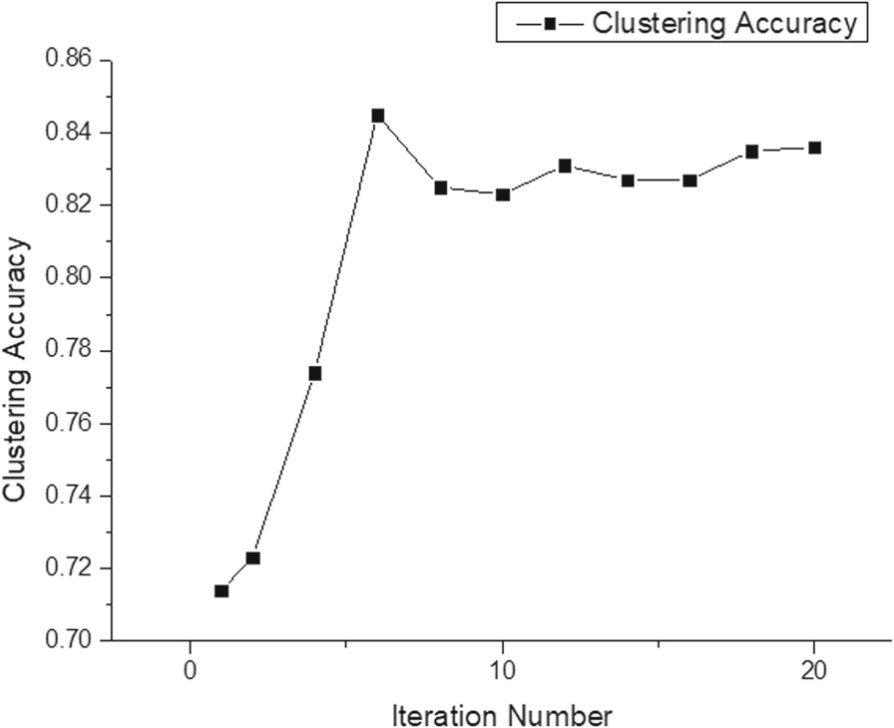
Clustering accuracy with different iteration numbers

We also examine the impact of iteration number on the clustering accuracy. As shown in Fig. 6, the clustering accuracy is poor when the iteration number is too small. As the iteration number becomes larger, the accuracy improves and then stabilizes.

**Fig. 7:**
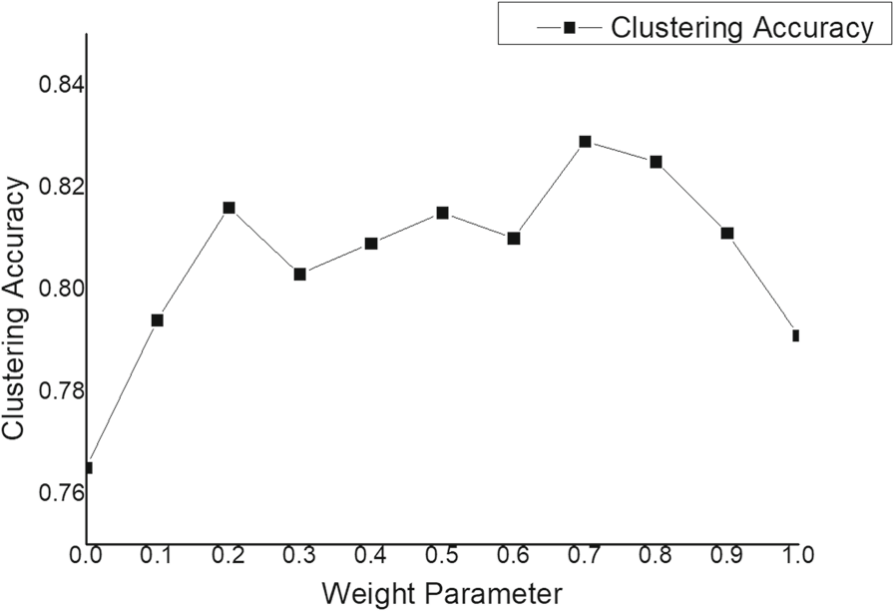
Clustering accuracy with different weight parameters

Lastly, we examine the impact of the weight parameter *α* and the result is shown in Fig. 7, If the weight parameter *α* is too small or too large, it means that we only consider one kind of meta path based relationships. Shorter meta paths have more information than longer ones. If *α*=1, the clustering accuracy equals 0.791, which is larger than 0.765(*α*=0).

### Qualitative evaluation

We apply our methods to investigate whether TCM-Clus can effectively cluster formulas into informative categories. The results are testified by TCM experts, and many of them are widely used in clinical diagnosis. We show the top-10 herbs and formulas in a cluster identified by our method in Table 2 and the top-5 functions and symptoms in a cluster in Table 3.

**Table 2:** Top-10 Herbs and Formulas in A Cluster

	Formula	Rank	Herb	Rank
1	(tian xing chi yan fang)	0.0199	(Chinese liquorice)	0.0612
2		0.0136	(weeping forsythia)	0.0604
3	(nei shu huang lian tang)	0.0128	(gardenia)	0.0592
4	(shu feng qing re tang)	0.0120	(A. propinquus)	0.0471
5	(xie fei yin)	0.0110	(Chinese peony root)	0.0425
6	(qing yan li ge tang)	0.0104	(Chinese goldthread)	0.0411
7	(liang ge san)	0.0102	(Chinese bellflower)	0.0353
8	(huan yin jiu ku tang)	0.0078	(female ginseng)	0.0288
9	(liang ying qing qi tang)	0.0076	(gypsum fibrosum)	0.0279
10	(qing wen bai du yin)	0.0072	(tenuifolia)	0.0274

**Table 3:** Top-5 Functions and Symptoms in A Cluster

	Function	Rank	Symptom	Rank
1	(clearing heat and detoxifying)	0.0213	(swelling and pain of eye)	0.0135
2	(dispelling wind and heat)	0.0207	(red face and labial coke)	0.0130
3	(clearing heat QI)	0.0185	(sore throat and losing voice)	0.0124
4	(cooling the blood and detoxifying)	0.0143	(coughing with lung heat)	0.0117
5	(clearing bowel and visceral heat)	0.0122	(feeling muggy and distension)	0.0106

### Case evaluation

As mentioned above, TCM-Clus can achieve high quality categorizations. Furthermore, we can obtain new knowledge from clusters, such as “different formulas with similar herbs”, “different formulas with similar functions”, “different symptoms with similar herbs” and so on. We show an example of “different symptoms with similar herbs” discovered by TCM-Clus in Table 4.

**Table 4:** Different Symptoms with Similar Herbs

Symptoms	Herbs	Common Herbs
(epigastric pain), (heartburn), (belching), (fullness) (tongue coating),	(red thorowax),(bitter orange), (Chinese peony), (Java grass), (Chuanxiong), (mandarine peel), (magnolia-bark), (barley), (Chinese liquorice), (rice sprout)	(red thorowax), (bitter orange), (Chinese peony), (Java grass), (Chuanxiong), (mandarine peel), (Chinese liquorice)
(Poor food and drink), (Fatigue), (stringy pulse), (pink tongue), (Liver distention and pain)	(red thorowax),(bitter orange),(Chinese peony), (Java grass),(Chuanxiong),(mandarine peel), (female ginseng),(woad root),(Field pennycress), (Chinese liquorice),(red dates)	
(pale tongue), (thin coating) (Live Qi), (stringy pulse), (Lump in breast)	(red thorowax),(bitter orange),(Chinese peony), (Java grass),(Chuanxiong),(mandarine peel), (rhizoma sparganii),(curcuma zedoary),(raw oyster), (Chinese liquorice),(sea-tent),(Sargassum)	

Besides, given a symptom as an input, our system can output proper herb/formula for the symptom. We have listed the herbs used for two symptoms in Table 5. The results are testified by TCM experts, and many of them are widely used in these symptoms.

**Table 5:** An example for our recommendation

Symptom	(pharyngitis)	(Deficiency of spleen and deficiency of food)
Herb 1	(Picria fel-terrae)	(sea-buckthorn)
Herb 2	(semen oroxyli)	(fuling)
Herb 3	(lignum et folium trachelospermi)	(ginseng)
Herb 4	(herba taching)	(yam)
Herb 5	(Lonicera confusa DC.)	(Chinese-date)

## Discussion

Based on our algorithm, we can learn potential knowledge in TCM, such as discovering similar prescriptions and recommending Chinese medicine based on symptoms. There are still some entities that we have not considered, such as the amount of herb and the information of patients. In our future work, more research is needed to address general HINs with more kinds of entities. In addition, the ranking function is highly related to different domains, and how we can automatically extract rules based on small partial ranking results given by experts could be another interesting problem.

## Conclusions

TCM is one of the most important complementary and alternative medicines. However, the complexity and elusiveness of diagnostic methods limit its development and generalization. Formulas are an essential part of TCM. Mining categorizations from TCM medical records is an important task for precision medicine. We present a novel algorithm, TCM-Clus, for mining formula categorization. We use a generative probabilistic model based on ranking to generate the reachable probability of target objects. Meanwhile, Bayesian rules and the EM algorithm are utilized to estimate the posterior probability. The experiments show that TCM-Clus achieves better clustering results than other representative algorithms and is beneficial for enhancing the predictive accuracy of medicine.

## Data Availability

The Chinese Pharmacopoeia 2015 Edition (http://wp.chp.org.cn/en/index.html).

## Abbreviations

HIN: Heterogeneous information network TCM: Traditional Chinese medicine
